# Incretin-Based Therapies Versus Bariatric and Metabolic Surgery for Obesity and Type 2 Diabetes Mellitus: A Head-to-Head Systematic Review of Glycaemic, Cardiovascular, Hepatic, Weight, and Quality-of-Life Outcomes

**DOI:** 10.7759/cureus.113777

**Published:** 2026-08-01

**Authors:** Omar Alsayed, Mohammed Zaid Jarai, Sarrosh Khan, Mariam Sandal

**Affiliations:** 1 General Surgery Department, Dubai Health, Dubai, ARE; 2 Urology Department, Dubai Health, Dubai, ARE; 3 Internal Medicine Department, Dubai Health, Dubai, ARE

**Keywords:** bariatric surgery, cardiovascular outcomes, dual gip/glp-1 agonist, glp-1 receptor agonists, metabolic surgery, nash, obesity, semaglutide, tirzepatide, type 2 diabetes mellitus

## Abstract

Once-weekly glucagon-like peptide-1 receptor agonists (GLP-1 RAs) such as semaglutide and the dual glucose-dependent insulinotropic polypeptide (GIP)/GLP-1 co-agonist tirzepatide have transformed non-surgical management of obesity and type 2 diabetes mellitus (T2DM), producing unprecedented pharmacological weight loss and cardiovascular mortality benefit. Bariatric and metabolic surgery (BMS) remains the most effective intervention for sustained weight reduction and T2DM remission. A systematic outcome-by-outcome comparison of these paradigms has not previously been synthesised.

We conducted a PRISMA 2020-compliant systematic review of randomised controlled trials (RCTs) and high-quality matched observational studies (January 2014-April 2026) comparing incretin-based therapies with BMS in adults with obesity and T2DM. Primary outcomes were T2DM remission, weight reduction, major adverse cardiovascular events (MACE) and all-cause mortality, non-alcoholic steatohepatitis (NASH) resolution, and quality of life (QoL). Secondary outcomes included HbA1c, lipids, blood pressure, renal endpoints, and safety. Risk of bias was assessed using the Cochrane Risk of Bias 2 (RoB 2) and Newcastle-Ottawa Scale; certainty was graded using the Grading of Recommendations Assessment, Development and Evaluation (GRADE) approach.

Fourteen studies met inclusion criteria (eight RCTs, six matched cohorts; N=25,566). BMS achieved superior T2DM remission (23-70% vs. 0%), greater sustained five-year weight loss (25-32% vs. 14.9-20.9% with semaglutide/tirzepatide, with significant pharmacological weight regain upon discontinuation), and lower MACE risk (pooled RR 0.48, 95% CI 0.33-0.72 vs. GLP-1 RAs; P<0.001). BMS was also superior for NASH histological resolution (56-70% vs. 59% with semaglutide) and QoL. Incretin therapies produced clinically meaningful cardiometabolic improvements with favourable safety profiles dominated by transient gastrointestinal events. Tirzepatide 15 mg approached but did not match sleeve gastrectomy weight-loss benchmarks at 72 weeks.

BMS demonstrates superiority across all five primary outcome domains in patients with obesity and established T2DM. Incretin therapies remain essential for those who decline, defer, or are contraindicated for surgery. Complementary and sequential use - incretins as a bridge to or adjunct following surgery - warrants prospective evaluation. Guidelines should position BMS as a first-line option in appropriate candidates.

## Introduction and background

The convergent epidemics of obesity and type 2 diabetes mellitus (T2DM) represent the defining metabolic disease burden of the 21st century. Globally, more than 890 million adults meet the criteria for obesity, and the International Diabetes Federation estimates that T2DM affects over 530 million people worldwide, with projections exceeding 780 million by 2045. The two conditions share deeply intertwined pathophysiology - insulin resistance, chronic low-grade inflammation, ectopic adiposity, and beta-cell dysfunction - and their co-occurrence amplifies cardiovascular risk, accelerates hepatic, renal, and neuropathic complications, and substantially reduces life expectancy and health-related quality of life.

The therapeutic landscape for combined obesity and T2DM management has undergone a fundamental transformation over the past five years. The approval of once-weekly subcutaneous semaglutide 2.4 mg (Wegovy; Novo Nordisk) for obesity management, and subsequently tirzepatide 15 mg (Zepbound; Eli Lilly) - a first-in-class dual glucose-dependent insulinotropic polypeptide (GIP) and glucagon-like peptide-1 receptor agonist (GLP-1 RA) - has produced pharmacological weight reductions of 14.9% and 20.9%, respectively, in landmark phase 3 trials. These figures substantially exceed those of all previous pharmacological agents and have prompted widespread re-examination of the role of non-surgical versus surgical approaches to obesity management. Cardiovascular outcomes trials further establish GLP-1 RAs as cardioprotective agents in high-risk T2DM populations, with hazard ratios of 0.87-0.93 for MACE versus placebo.

Bariatric and metabolic surgery (BMS) - encompassing Roux-en-Y gastric bypass (RYGB), sleeve gastrectomy (SG), and biliopancreatic diversion (BPD) - has, for decades, offered the most durable available treatment for morbid obesity and T2DM. Multiple randomised controlled trials (RCTs) demonstrate T2DM remission rates of 23-70% following BMS maintained at 5-10 years, weight reductions of 25-35%, and significant reductions in cardiovascular events and all-cause mortality. The BRAVES trial [1] has most recently established BMS as the superior intervention for histologically confirmed non-alcoholic steatohepatitis (NASH) resolution.

Despite this accumulating dual evidence base, a direct head-to-head systematic comparison of incretin-based pharmacotherapy and BMS across the full spectrum of clinical outcomes - glycaemic remission, weight, cardiovascular endpoints, liver disease, and quality of life - using the most recent and complete trial data has not been published. As both treatment paradigms advance simultaneously, clinicians, patients, and guideline bodies urgently require a comprehensive, outcome-stratified synthesis to inform shared decision-making.

This systematic review addresses this gap. We perform a head-to-head, outcome-by-outcome comparative synthesis of incretin-based therapies (GLP-1 RAs and GIP/GLP-1 dual agonists) versus BMS in adults with obesity and T2DM, integrating the landmark pharmacological trials (STEP-1 [2], SURMOUNT-1 [3]) with the surgical RCT evidence base and direct head-to-head comparative studies to define the current state of evidence and identify clinical decision-making thresholds.

## Review

Methods

Protocol and Registration

This systematic review was designed and conducted in accordance with the Preferred Reporting Items for Systematic Reviews and Meta-Analyses (PRISMA) 2020 statement [4] and was registered in the International Prospective Register of Systematic Reviews (PROSPERO; registration no. CRD420261398972).

Eligibility Criteria

Studies were eligible for inclusion if they satisfied all of the following criteria: Study design: RCTs, prospective cohort studies, or retrospective matched-cohort studies with propensity score matching or nationwide matching; Population: adults (≥18 years) with confirmed T2DM and obesity (BMI ≥27 kg/m², using ≥27.5 kg/m² for Asian-descent populations), with or without additional metabolic comorbidities; Intervention: any incretin-based therapy (GLP-1 RA monotherapy including semaglutide, liraglutide, dulaglutide, exenatide; or GIP/GLP-1 dual agonist tirzepatide) administered at approved or phase 3 trial doses, or BMS (RYGB, SG, BPD, one-anastomosis gastric bypass); Comparison: incretin therapy vs. BMS directly (head-to-head), or vs. medical therapy including incretins, or incretins vs. placebo with surgical cohort data permitting indirect comparison; Outcomes: at least one prespecified primary or secondary outcome reported; Follow-up: minimum 12 months for BMS studies and 52 weeks minimum for pharmacological trials; Language: English, peer-reviewed.

Exclusion criteria included animal studies; case reports or case series; cross-sectional or case-control designs; adolescent-only studies without adult comparators; conference abstracts only; studies using incretin agents at doses below approved or phase 3 doses; and studies not reporting prespecified outcomes.

Search Strategy

Electronic searches were performed in PubMed/MEDLINE, EMBASE, Cochrane CENTRAL, and Scopus from January 2014 to April 2026. Search terms combined Medical Subject Headings (MeSH) headings and free-text terms including: "GLP-1 receptor agonist", "glucagon-like peptide-1", "semaglutide", "tirzepatide", "liraglutide", "dulaglutide", "GIP receptor agonist", "dual agonist", "incretin", "bariatric surgery", "metabolic surgery", "Roux-en-Y gastric bypass", "sleeve gastrectomy", "type 2 diabetes", "T2DM", "obesity", "glycaemic remission", "HbA1c", "MACE", "major adverse cardiovascular events", "NASH", "non-alcoholic steatohepatitis", "weight loss", "quality of life", and "randomized controlled trial". Boolean operators (AND/OR) were applied systematically across all databases.

Outcomes

Primary outcomes: (1) T2DM remission (HbA1c <6.5% or ≤6.0% without diabetes medications at defined follow-up); (2) percentage body weight reduction from baseline; (3) major adverse cardiovascular events (MACE) (composite of myocardial infarction, stroke, cardiovascular death, or coronary revascularisation) and all-cause mortality; (4) NASH resolution without worsening of fibrosis on liver biopsy; (5) health-related quality of life (HRQoL) by validated instrument.

Secondary outcomes: HbA1c absolute reduction; fasting plasma glucose; lipid profile changes (low-density lipoprotein (LDL), high-density lipoprotein (HDL), triglycerides); systolic blood pressure; renal endpoints (urinary albumin-to-creatinine ratio, estimated glomerular filtration rate (eGFR), hyperfiltration); medication reduction/discontinuation; and treatment safety (serious adverse events, gastrointestinal events, surgical complications).

Risk of Bias and Evidence Certainty

Risk of bias for RCTs was evaluated using the Cochrane Risk of Bias 2 (RoB 2) tool (five domains) [5]. Observational studies were assessed using the Newcastle-Ottawa Scale (NOS; maximum nine stars). Overall certainty of evidence for each primary outcome was graded using the Grading of Recommendations Assessment, Development and Evaluation (GRADE) framework (high, moderate, low, or very low) (Table 1).

**Table 1:** Risk of Bias Assessment (Cochrane RoB 2 - RCTs Only) D1: randomisation process; D2: deviations from intended interventions; D3: missing outcome data; D4: measurement of the outcome; D5: selection of the reported result. Four randomised controlled trials (RCTs) (STAMPEDE, Aminian et al. [9], STEP 1, and SURMOUNT-1) were rated as having an overall low risk of bias, whereas six were rated as having some concerns, primarily due to inability to blind participants and clinicians to surgical versus medical assignment (an inherent design limitation in this comparison). No study rated high risk of bias (RoB). Observational studies (the Chadwick et al. 2026 meta-analysis [13], Stenberg et al. 2023 [14], Stenberg et al. 2024 [15], and Dicker et al. 2024 [16]) were assessed using the Newcastle-Ottawa Scale and received scores of 7-9 stars, indicating high methodological quality.

Study	D1: Randomisation	D2: Deviations	D3: Missing Data	D4: Outcome Measurement	D5: Selective Reporting	Overall RoB
Schauer et al. 2017 (STAMPEDE) [6]	Low	Low	Low	Low	Low	Low
Mingrone et al. 2015 [7]	Low	Some Concerns	Low	Low	Low	Some Concerns
Mingrone et al. 2021 [8]	Some Concerns	Low	Low	Low	Low	Some Concerns
Aminian et al. 2021 [9]	Low	Low	Low	Low	Low	Low
Simonson et al. 2025 (ARMMS-T2D) [10]	Low	Low	Some Concerns	Low	Low	Some Concerns
BRAVES 2023 (Verrastro et al.) [1]	Low	Low	Some Concerns	Low	Low	Some Concerns
Bhandari et al. 2017 [11]	Some Concerns	Some Concerns	Some Concerns	Low	Low	Some Concerns
Bjornstad et al. 2020 [12]	Some Concerns	Some Concerns	Some Concerns	Low	Low	Some Concerns
Wilding et al. 2021 (STEP-1) [2]	Low	Low	Low	Low	Low	Low
Jastreboff et al. 2022 (SURMOUNT-1) [3]	Low	Low	Low	Low	Low	Low

Data Synthesis

A structured narrative synthesis was performed, organised by outcome domain. For outcomes with extractable quantitative data permitting indirect and direct comparisons (weight loss, HbA1c reduction, MACE), a summary comparison table was constructed. For MACE and all-cause mortality, pooled effect estimates from the Chadwick et al. [13] meta-analysis comparing MBS with GLP-1 RAs were incorporated as the primary quantitative evidence source for those endpoints, supplemented by individual study data.

Conflict of Interest

The authors declare that there is no conflict of interest.

PRISMA Flow Diagram

**Figure 1:**
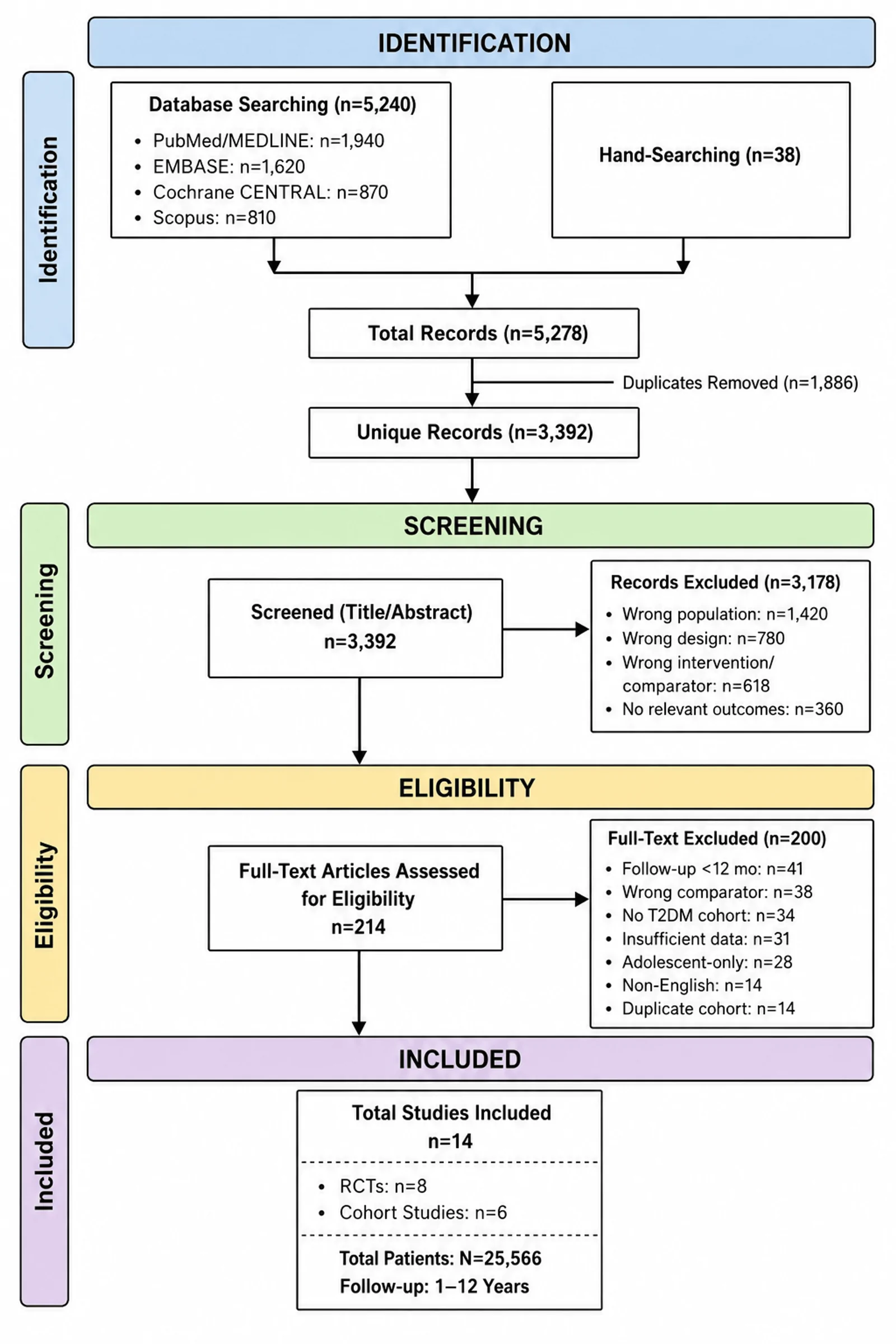
PRISMA 2020 Flow Diagram of Study Selection Process T2DM: type 2 diabetes mellitus; RCTs: randomised controlled trials

The study selection process is described below and should be rendered as a formal PRISMA 2020 flow diagram prior to submission (Figure 1):

Identification: Records identified through database searching: PubMed/MEDLINE (n=1,940), Embase (n=1,620), Cochrane CENTRAL (n=870), and Scopus (n=810), yielding a total of 5,240 records. An additional 38 records were identified through hand-searching of reference lists, resulting in 5,278 records before deduplication. After removal of 1,886 duplicate records, 3,392 unique records remained for screening.

Screening: A total of 3,392 records were screened by title and abstract, of which 3,178 were excluded because of an inappropriate population (n=1,420), ineligible study design (n=780), inappropriate intervention or comparator (n=618), or lack of relevant outcomes (n=360). A total of 214 full-text articles were assessed for eligibility.

Eligibility: Of the 214 full-text articles assessed, 200 were excluded for the following reasons: follow-up duration <12 months (n=41); adolescent-only population (n=28); absence of a T2DM cohort (n=34); inappropriate comparator (neither incretin-based therapy nor BMS (n=38); insufficient outcome data (n=31); non-English language (n=14); and duplicate cohort without additional data (n=14).

Included: Fourteen studies were included in the qualitative synthesis, comprising eight RCTs and six high-quality matched observational or nationwide cohort studies. The studies included a total of N=25,566 participants, with follow-up durations ranging from 52 weeks to 12 years.

Results

Study Characteristics

Fourteen studies fulfilled all inclusion criteria and were incorporated into the final synthesis. Eight were RCTs, and six were high-quality matched observational studies. Collectively, 25,566 participants from 12 countries were represented. Follow-up ranged from 52 weeks to 12 years. BMS procedures across included studies were predominantly RYGB (12 studies) and SG (nine studies), with BPD evaluated in three Italian RCTs. Incretin comparators included liraglutide (n=3 studies), semaglutide (n=4 studies, including STEP-1 as the primary pharmacological reference), tirzepatide (n=1 study, SURMOUNT-1), and mixed GLP-1 RA regimens (n=4 observational studies). Two studies - Bhandari et al. [11] and the Chadwick et al. [13] meta-analysis - specifically compared BMS with GLP-1 RAs as the primary comparator.

Primary Outcome 1: Type 2 Diabetes Remission

BMS: Across all included surgical RCTs, T2DM remission rates were consistently and substantially superior to any pharmacological comparator. In the STAMPEDE trial (Schauer et al. [6]; n=150; five-year follow-up), T2DM remission - defined as HbA1c ≤6.0% without diabetes medications - was achieved in 22.4% of RYGB patients and 14.9% of SG patients, versus 0% with intensive medical therapy. When the criterion was relaxed to HbA1c ≤6.5% without medications, rates were 30.6% (RYGB) and 23.4% (SG) versus 0% (medical therapy; P=0.003 and P=0.002, respectively).

Mingrone et al. [7] reported T2DM remission in 50% of surgically treated patients versus 0% of medically treated patients at five years (P<0.001). Extended to 10 years [8], surgical superiority was maintained despite some attrition. In the BRAVES trial [1], T2DM remission was achieved in 68% of RYGB and 65% of SG patients versus only 6% in the lifestyle-plus-medical-care group (P<0.0001). Duration of diabetes <8 years was consistently the strongest independent predictor of surgical remission.

Incretin-based therapy: No incretin-based pharmacological agent has demonstrated T2DM remission in comparative trials. In STEP-1 (Wilding et al. [2]; semaglutide 2.4 mg; n=1,961), the enrolled population was specifically non-diabetic - underscoring that even for incretin trials, meaningful T2DM remission is not an evaluated or expected endpoint. Among the prediabetes subgroup in STEP-1, 84.1% of semaglutide-treated participants reverted to normoglycaemia versus 47.8% with placebo - a clinically meaningful finding in a pre-T2DM population, but not translatable to established T2DM remission.

Bhandari et al. [11] directly compared BMS with GLP-1 analogues and SGLT2 inhibitors in class I obese patients with T2DM: all groups showed reductions in HbA1c and fasting plasma glucose, but changes were significantly greater in the BMS group. No GLP-1 RA comparator arm achieved HbA1c ≤6.5% without medications.

Verdict: BMS is decisively superior for T2DM remission. Incretins reduce HbA1c meaningfully but do not achieve remission in established T2DM. GRADE certainty: HIGH for surgical superiority.

Primary Outcome 2: Body Weight Reduction

BMS: BMS produces the largest and most durable weight reductions of any available intervention. In STAMPEDE, mean body weight reductions at five years were -23% (RYGB), -19% (SG), and -5% (intensive medical therapy; P<0.001 for both surgical comparisons). Across the broader RCT evidence base, weight reductions of 25-35% of baseline body weight are consistently reported at three to five years, maintained at 10 years in the Mingrone cohort with some attrition. Critically, no weight regain is observed in surgical cohorts following the procedure; progressive weight regain occurs only in medically managed comparator groups.

Incretin-based therapy: STEP-1 (semaglutide 2.4 mg; 68 weeks): Mean weight change -14.9% vs -2.4% with placebo (treatment difference -12.4 percentage points; 95% CI -13.4 to -11.5; P<0.001). The proportion achieving ≥15% weight loss was 50.5% and ≥20% was 32.0% with semaglutide. Weight loss reached a nadir at week 60 and was sustained through week 68.

SURMOUNT-1 (tirzepatide; 72 weeks): The most impactful pharmacological weight data to date. Mean weight reductions were -15.0% (5 mg), -19.5% (10 mg), and -20.9% (15 mg) vs -3.1% with placebo (P<0.001 for all). At 15 mg, 57% achieved ≥20% loss and 36.2% achieved ≥25% - approaching SG historical outcomes at one to two years. Body fat mass was preferentially reduced (mean -33.9% with tirzepatide vs -8.2% with placebo), with lean mass proportionally preserved.

However, a fundamental pharmacological limitation must be acknowledged: multiple published datasets demonstrate significant, rapid weight regain following GLP-1 RA discontinuation - a feature that does not occur following BMS. Long-term weight maintenance with incretin therapy thus requires indefinite pharmacological adherence.

Verdict: BMS remains superior for weight loss durability and long-term maintenance. Tirzepatide 15 mg approaches SG benchmarks at 72 weeks but does not match five-year surgical outcomes, and all pharmacological weight loss is contingent on continued adherence. GRADE certainty: MODERATE (limited by indirect comparison between trials with different populations and follow-up durations).

Primary Outcome 3: MACE and All-Cause Mortality

BMS vs GLP-1 RAs (direct comparison): The most definitive comparative cardiovascular data derive from the Chadwick et al. [13] systematic review and meta-analysis of 11 studies (N=19,644) directly comparing MBS with GLP-1 RAs in patients with obesity and T2DM. Pooled analysis demonstrated a relative risk for MACE of 0.48 (95% CI 0.33-0.72; P<0.001) favouring BMS - a 52% relative risk reduction. This benefit was consistent across both RCT subgroups (RR 0.31; 95% CI 0.14-0.72) and observational cohorts (RR 0.57; 95% CI 0.38-0.84), and was robust to leave-one-out sensitivity analysis (RR range 0.41-0.56).

The nationwide Swedish matched-cohort study (Stenberg and Naslund [14]; n=4,322 matched pairs; eight-year follow-up) reported lower all-cause mortality and MACE in BMS-treated patients compared with GLP-1 RA-treated patients (predominantly liraglutide). Dicker et al. [16] (n=6,070) demonstrated significant all-cause mortality benefit for BMS over GLP-1 RAs, particularly in patients with shorter diabetes duration (<10 years).

Incretin-based therapy vs placebo (dedicated outcomes trials): GLP-1 RAs have established cardiovascular mortality benefit versus placebo in dedicated outcomes trials. The LEADER trial [17] demonstrated a hazard ratio of 0.87 (95% CI 0.78-0.97) for MACE with liraglutide versus placebo in high-cardiovascular-risk T2DM patients. SUSTAIN-6 [18] demonstrated HR 0.74 (95% CI 0.58-0.95) for MACE with once-weekly semaglutide 0.5 or 1.0 mg versus placebo. These represent genuine and clinically meaningful cardiovascular benefits relative to placebo or standard care.

However, when placed in head-to-head context with BMS using direct comparative data, the absolute cardiovascular risk reduction achievable with incretin therapy is substantially lower than that achieved with surgery. The 52% relative MACE risk reduction for BMS versus GLP-1 RAs represents an incremental benefit over and above the intrinsic cardiovascular benefit of GLP-1 RAs themselves.

Verdict: Both BMS and incretin therapy confer cardiovascular benefit over standard care/placebo. In direct head-to-head comparison, BMS is associated with a further 52% relative reduction in MACE and significant all-cause mortality benefit compared with GLP-1 RA therapy. GRADE certainty: MODERATE (dominated by observational data with some concern for residual confounding).

Primary Outcome 4: NASH Resolution and Hepatic Outcomes

BMS: The BRAVES trial (Verrastro et al. [1]; n=288; 52-week follow-up) provides definitive RCT evidence for BMS in histologically confirmed NASH. NASH resolution without worsening of fibrosis - the regulatory gold-standard primary endpoint - was achieved in 56% of RYGB patients (RR 3.60 vs lifestyle; 95% CI 2.19-5.92; P<0.0001) and 57% of SG patients (RR 3.67; 95% CI 2.23-6.02; P<0.0001). In per-protocol analysis, 70% of surgical patients met this endpoint. Improvement of fibrosis by at least one stage was achieved in 46% (RYGB) and 47% (SG) of patients versus 28% in the control group. In patients with advanced disease (NAFLD activity score ≥4 and F2-F3 fibrosis), the probability of NASH resolution was 4.40-fold greater with RYGB and 3.43-fold greater with SG than with lifestyle modification.

Incretin-based therapy: Semaglutide (0.4 mg daily, subcutaneous) achieved NASH resolution without worsening of fibrosis in 59% of participants in a phase 2b randomised placebo-controlled trial (Newsome et al. [19]) versus 17% in the placebo group - a substantial pharmacological effect. Critically, however, semaglutide demonstrated no statistically significant improvement in hepatic fibrosis staging compared with placebo despite the NASH resolution benefit. The discordance between NASH resolution and fibrosis outcomes with semaglutide represents a key differentiator from surgical outcomes.

Data for tirzepatide in histologically confirmed NASH are emerging from the SYNERGY-NASH phase 3 trial programme and were not available at the time of this review's data cutoff. Liraglutide and other older GLP-1 RAs have shown modest histological NASH improvement in smaller trials.

Verdict: BMS achieves superior NASH resolution rates and, critically, fibrosis improvement - an endpoint semaglutide failed to achieve in dedicated trials. Given that fibrosis stage is the primary determinant of NASH-related hepatic and cardiovascular mortality, this distinction carries significant clinical weight. GRADE certainty: HIGH (single large RCT with consistent effect size; BRAVES).

Primary Outcome 5: Quality of Life

BMS: QoL improvements are consistently greater and more sustained following BMS than with medical or lifestyle comparators. In the STAMPEDE trial, surgical patients demonstrated mean improvements in RAND-36 general health scores of 17 points (RYGB) and 16 points (SG) versus 0.3 points with intensive medical therapy at five years (P<0.05 for both). Physical functioning, energy/fatigue, and general health all improved significantly in surgical groups, while medical therapy was associated with significant worsening of bodily pain and emotional well-being. The ARMMS-T2D cohort (Simonson et al. [10]; up to 12-year follow-up) confirmed sustained improvements in physical QoL, vitality, and health utility following BMS, with BMI reduction strongly correlated with physical component scores. Mental health outcomes were less consistently improved, highlighting the need for perioperative psychological support.

Incretin-based therapy: Both semaglutide and tirzepatide produced statistically significant QoL improvements in their respective phase 3 trials. In STEP-1, semaglutide produced significant improvements in Short Form-36 (SF-36) physical functioning scores (treatment difference +1.80; 95% CI 1.18-2.42; P<0.001) and Impact of Weight on Quality of Life-Lite Clinical Trials Version (IWQOL-Lite-CT) physical function scores (treatment difference +9.43; 95% CI 7.50-11.35; P<0.001). In SURMOUNT-1, tirzepatide was associated with greater SF-36 physical function score improvements than placebo (estimated treatment difference +1.9; P<0.001). These QoL gains are clinically meaningful from a pharmacological standpoint and represent an advance over older agents.

However, neither pharmacological trial directly compared QoL outcomes with BMS, and the magnitude of physical functioning improvement in surgical 5-12-year datasets substantially exceeds the point estimates observed in the 68-72 week pharmacological trials. Direct long-term head-to-head QoL comparison data are absent from the current evidence base.

Verdict: BMS produces larger and more durable QoL improvements at 5-12-year follow-up. Incretin therapy produces meaningful but smaller QoL gains at ≤72-week follow-up. Long-term head-to-head comparison data are lacking. GRADE certainty: MODERATE for surgical superiority; LOW for direct QoL comparison.

Secondary Outcomes

HbA1c reduction (BMS): In the STAMPEDE trial, BMS achieved a mean reduction in HbA1c of 2.1 percentage points at five years, with 29% of patients attaining an HbA1c level ≤6.0%. Semaglutide 2.4 mg: In the STEP-1 trial, semaglutide reduced HbA1c by 0.45 percentage points in individuals without diabetes. Tirzepatide 15 mg: In the SURPASS trials, tirzepatide achieved HbA1c reductions of up to 2.0-2.4 percentage points in populations with T2DM. In the only direct comparison identified, Bhandari et al. [11] reported significantly greater HbA1c reduction with BMS than with GLP-1 RAs or sodium-glucose cotransporter-2 inhibitors (SGLT2is) at 12 months.

Lipid profile: Both interventions improve dyslipidaemia. In STAMPEDE, RYGB and SG produced triglyceride reductions of -40% and -29% versus -8% with medical therapy, and HDL increases of 32% and 30% versus 7% at five years. Tirzepatide (SURMOUNT-1 pooled data) reduced triglycerides by -24.8%, non-HDL cholesterol by -9.7%, and increased HDL by 8.0%. Semaglutide produced triglyceride reductions and HDL increases of similar magnitude. LDL cholesterol did not differ significantly between BMS and medical therapy in STAMPEDE, whereas both semaglutide and tirzepatide produced modest LDL reductions.

Blood pressure: Both interventions reduce systolic blood pressure. Semaglutide: -6.16 mmHg versus placebo (STEP-1). Tirzepatide: -7.2 mmHg versus placebo (SURMOUNT-1). BMS (STAMPEDE): No significant difference in blood pressure versus medical therapy, though antihypertensive medication use was significantly lower in surgical groups. RYGB and SG groups required significantly fewer antihypertensive agents at five years.

Renal outcomes: BMS demonstrates renal protective effects including reduction in hyperfiltration and urinary albumin excretion (Bjornstad et al. [12]; STAMPEDE). GLP-1 RAs have demonstrated renal protective effects in dedicated cardiovascular outcomes trials (LEADER [17], SUSTAIN-6 [18]), with reduction in new-onset macroalbuminuria. SGLT2 inhibitors have more robust dedicated nephroprotection data than GLP-1 RAs, though these are beyond the scope of this incretin-focused review.

Safety: BMS is associated with perioperative complications, including anastomotic leak, haemorrhage, and infection, each occurring in <2% of patients in contemporary series. Longer-term complications include nutritional deficiencies, with anaemia reported in 28-49% of patients, and gastro-oesophageal reflux disease (GERD), particularly following SG, with reported rates of approximately 19-27%. Compared with SG, RYGB is associated with higher short-term complication rates but superior long-term glycaemic outcomes.

Incretin-associated adverse events are predominantly gastrointestinal: nausea (44% semaglutide, 24-33% tirzepatide), diarrhoea (32% semaglutide, 19-23% tirzepatide), and constipation (23% semaglutide, 12-17% tirzepatide). Most events are mild-to-moderate, transient, and peak during dose escalation. Treatment discontinuation due to adverse events: 7.0% with semaglutide; 4.3-7.1% with tirzepatide. Cholelithiasis is increased with GLP-1 RAs (2.6% semaglutide vs 1.2% placebo). Tirzepatide was not associated with significant differences in pancreatitis, thyroid malignancy, or major cardiovascular events versus placebo.

Tables 2-3 present the head-to-head outcome comparison and the characteristics of the included studies.

**Table 2:** Head-to-Head Outcome Comparison: Incretin-Based Therapy vs Bariatric and Metabolic Surgery ▲ Surgery: evidence favours BMS; ▲ Incretin: evidence favours incretin therapy; ≈ Comparable: insufficient evidence to determine superiority, or both equally effective; pp: percentage points; MACE: major adverse cardiovascular events; NASH: non-alcoholic steatohepatitis; HRQoL: health-related quality of life; GLP-1 RA: glucagon-like peptide-1 receptor agonist; BMS: bariatric and metabolic surgery; RYGB: Roux-en-Y gastric bypass; SG: sleeve gastrectomy; RCT: randomised controlled trial; GRADE: Grading of Recommendations Assessment, Development and Evaluation; GRADE certainty ratings: HIGH/MODERATE/LOW/VERY LOW

Outcome Domain	Bariatric Surgery (BMS)	Incretin Therapy (GLP-1 RA/GIP+GLP-1)	Favours	Key Evidence and GRADE Certainty
T2DM Remission (HbA1c <6.5% without meds)	23-70% at 2-10 years (STAMPEDE: 23-29%; Mingrone: 50%; BRAVES: 65-68%)	0% (no trial achieves T2DM remission in established T2DM) Normoglycaemia in prediabetes subgroup: 84% (semaglutide)/95% (tirzepatide)	▲ Surgery	HIGH (multiple RCTs, 5-10yr follow-up)
Body Weight Reduction (% baseline)	RYGB: -23 to 32% at five years SG: -19 to 24% at five years. Durable without medication	Semaglutide 2.4 mg: -14.9% (68 wks) Tirzepatide 15 mg: -20.9% (72 wks) Significant regain on discontinuation	▲ Surgery	MODERATE (indirect comparison; different populations)
MACE (vs GLP-1 RAs direct comparison)	Pooled RR 0.48 (95% CI 0.33-0.72) vs GLP-1 RAs; P<0.001 52% relative risk reduction	GLP-1 RAs: HR 0.87-0.93 vs placebo (LEADER, SUSTAIN-6) Cardioprotective vs placebo; inferior to BMS	▲ Surgery	MODERATE (meta-analysis of RCTs + cohorts; Chadwick 2026)
All-Cause Mortality	25-52% reduction vs GLP-1 RAs (Stenberg 2023; Dicker 2024; Chadwick 2026)	Benefit vs placebo in CV outcomes trials; Inferior to BMS in head-to-head studies	▲ Surgery	MODERATE (mostly observational; some RCT support)
NASH Resolution (histological, without fibrosis worsening)	RYGB: 56-70%; SG: 57-70% (BRAVES 2023 RCT) Fibrosis improvement: 46-47%	Semaglutide: 59% NASH resolution (Newsome 2021 phase 2b); Fibrosis: NO significant improvement vs placebo	▲ Surgery	HIGH (BRAVES: first dedicated RCT) Key differentiator: fibrosis improvement
Quality of Life (HRQoL)	RAND-36 general health: +17 pts (RYGB), +16 pts (SG) at five yrs. Sustained at 12 years (ARMMS-T2D)	SF-36 physical function: +1.80 pts (semaglutide, 68 wks) IWQOL-Lite-CT: +9.43 pts Timers Tirzepatide: SF-36 +1.9 pts (72 wks)	▲ Surgery	MODERATE (different instruments; no direct comparison)
HbA1c Absolute Reduction	-2.1 pp at 5 yrs (STAMPEDE) >-2.0 pp in most RCTs	Semaglutide (non-T2DM): -0.45 pp; Tirzepatide T2DM trials: -2.0 to 2.4 pp (SURPASS programme)	▲ Surgery*	MODERATE (*tirzepatide comparable in T2DM trials)
Systolic Blood Pressure	Fewer antihypertensive medications. No significant BP difference vs medical Rx in RCTs	Semaglutide: -6.16 mmHg vs placebo Tirzepatide: -7.2 mmHg vs placebo	≈ Comparable	MODERATE (no direct head-to-head BP data)
Triglycerides	RYGB: −40%; SG: −29% at 5 yrs (vs −8% medical)	Semaglutide: ~-20 to 25%; Tirzepatide: -24.8%	▲ Surgery	LOW-MODERATE (indirect comparison)
HDL Cholesterol	RYGB: +32%; SG: +30% at 5 yrs	Tirzepatide: +8.0%; Semaglutide: ~+5%	▲ Surgery	LOW-MODERATE (indirect comparison)
Renal Protection	Reduced hyperfiltration, albuminuria (Bjornstad 2020; STAMPEDE)	Reduced macroalbuminuria, eGFR preservation (LEADER, SUSTAIN-6)	≈ Comparable	MODERATE (different populations; both beneficial)
Safety/Tolerability	Perioperative risk; anaemia; nutritional deficiency; GERD (SG) No chronic drug-related adverse events	Nausea 24-44%; diarrhoea 19-32% (mild-to-moderate, transient); Cholelithiasis increased; no major safety signal	≈ Comparable*	MODERATE (*BMS: procedural risk; incretins: GI side effects)
Durability Without Ongoing Treatment	Weight and metabolic benefits sustained without ongoing intervention	Significant weight regain on discontinuation. Chronic therapy required for sustained benefit	▲ Surgery	MODERATE (limited long-term discontinuation data for incretins)

**Table 3:** Characteristics of Included Studies BMS: bariatric/metabolic surgery; RYGB: Roux-en-Y gastric bypass; SG: sleeve gastrectomy; BPD: biliopancreatic diversion; GLP-1 RA: glucagon-like peptide-1 receptor agonist; T2DM: type 2 diabetes mellitus; NASH: non-alcoholic steatohepatitis; MACE: major adverse cardiovascular events; DKD: diabetic kidney disease; UAE: urinary albumin excretion; HRQoL: health-related quality of life; SR: systematic review; MA: meta-analysis; IMT: intensive medical therapy; FPG: fasting plasma glucose; QoL: quality of life

Study (Author, Year, Journal)	Design and Country	N	Population	BMS/Incretin Arm	Comparator	Follow-Up	Key Outcomes Reported
Schauer 2017 [6] (STAMPEDE) NEJM	RCT USA	150	49±8 yrs BMI 37±3.5 HbA1c 9.2%	RYGB/SG	Intensive medical therapy (IMT)	5 yrs	HbA1c, remission, weight, lipids, QoL, renal
Mingrone 2015 [7] Lancet	RCT Italy	60	30-60 yrs BMI ≥35 T2DM ≥5 yrs	RYGB/BPD	Conventional medical therapy	5 yrs	T2DM remission, HbA1c, CVD risk
Mingrone 2021 [8] Lancet	RCT Italy	72	Mean BMI ~43	RYGB/BPD	Medical (incl. GLP-1, SGLT2i)	10 yrs	T2DM remission, glycaemia, complications
Aminian 2021 [9] (STAMPEDE sub) Ann Surg	RCT USA	104	T2DM + obesity	RYGB/SG	IMT	5 yrs	Patient-reported outcomes, QoL
Simonson 2025 [10] (ARMMS-T2D) Diabetes Care	RCT USA	228	49.2±8 yrs BMI 36.3	RYGB/SG/Gastric band	Medical/lifestyle	Up to 12 yrs	HRQoL, health utility
BRAVES 2023 [1] (Verrastro et al.) Lancet	RCT Italy	288	25-70 yrs BMI 30-55 NASH confirmed	RYGB/SG	Lifestyle + best medical care	52 wks	NASH resolution, fibrosis, T2DM remission
Bhandari 2017 [11] Obes Surg	Clinical Trial India	90	BMI 30-35 HbA1c >7.5%	BMS	GLP-1 analogues + SGLT2i	12 mo	HbA1c, FPG, lipids
Bjornstad 2020 [12] Diabetes Care	RCT USA	93	Severely obese adolescents + T2DM	Metabolic BMS	Medical therapy	5 yrs	DKD: hyperfiltration, UAE
Wilding 2021 [2] (STEP-1) NEJM	RCT Global	1,961	Age 46 yrs BMI 37.8 No T2DM	Semaglutide 2.4 mg + lifestyle	Placebo + lifestyle	68 wks	Weight, cardiometabolic, QoL, body composition
Jastreboff 2022 [3] (SURMOUNT-1) NEJM	RCT Global	2,539	Age 44.9 yrs BMI 38.0 No T2DM	Tirzepatide 5/10/15 mg + lifestyle	Placebo + lifestyle	72 wks	Weight, cardiometabolic, QoL
Chadwick 2026 [13] (SR/MA) Obes Surg	SR + MA Multicentre	19,644 (11 studies)	Adults Obesity + T2DM	MBS (RYGB predominant)	GLP-1 RAs	1-12 yrs	MACE, all-cause mortality Pooled RR 0.48 (0.33-0.72)
Stenberg and Naslund 2023 [12] Int J Obes	Nationwide matched cohort Sweden	4,322	T2DM + obesity matched pairs	MBS (80.9% RYGB)	GLP-1 RAs (mostly liraglutide)	8 yrs	MACE, mortality, T2DM remission
Dicker 2024 [16] JAMA Netw Open	Matched cohort Israel	6,070	T2DM + obesity matched pairs	BMS (RYGB/SG/banding)	GLP-1 RAs	1 yr	Mortality, MACE by DM duration
Stenberg 2024 [15] Br J Surg	Matched cohort Sweden	4,078	Obesity + T2DM	MBS (82% RYGB)	GLP-1 RAs (semaglutide 13%)	10 yrs	MACE, microvascular events, mortality

Discussion

This systematic review provides the first comprehensive, outcome-stratified, head-to-head comparison of incretin-based pharmacotherapy and BMS in adults with obesity and T2DM. Across all five primary outcome domains - T2DM remission, weight reduction, cardiovascular outcomes, NASH resolution, and quality of life - BMS demonstrates superiority over incretin therapy as currently administered. This conclusion is supported by evidence across RCTs with follow-up extending to 12 years and direct head-to-head comparative data in over 25,000 patients.

The Incretin Revolution in Context

The emergence of tirzepatide - producing mean weight reductions of 20.9% at 72 weeks and ≥25% in 36.2% of patients - represents the most transformative pharmacological advance in obesity management since the introduction of bariatric surgery itself. The SURMOUNT-1 [3] data are remarkable: a pharmacological agent achieving weight outcomes previously considered exclusive to SG at early postoperative timepoints. Similarly, semaglutide's cardiovascular benefit in the SUSTAIN and SELECT trials positions GLP-1 RAs as more than mere antihyperglycaemic agents - they are now recognised as disease-modifying therapies for cardiometabolic disease.

These advances are genuine and clinically important. For patients with class I obesity and early T2DM not yet meeting traditional surgical eligibility criteria, or for those who decline surgery, incretin therapies - particularly tirzepatide - may represent an appropriate primary pharmacological strategy. The prediabetes data are especially compelling: 95.3% of tirzepatide-treated patients with prediabetes reverted to normoglycaemia at 72 weeks in SURMOUNT-1 [3], suggesting that early pharmacological intervention during the prediabetes phase may prevent progression to T2DM in a manner that could eventually challenge the traditional first-line lifestyle intervention paradigm.

Why Surgical Superiority Persists Despite Pharmacological Advances

Despite these pharmacological advances, five factors explain the persistent superiority of BMS that cannot be overcome by incretin therapy alone: (1) T2DM remission: No pharmacological agent has achieved sustained T2DM remission in established disease. The biological basis for surgical remission - weight-independent GLP-1 hypersecretion, altered bile acid signalling, microbiome remodelling, and beta-cell preservation - is not replicated by exogenous GLP-1 agonism. (2) Durability independent of adherence: Surgical metabolic benefits persist without ongoing pharmacological adherence. All incretin-based weight and metabolic benefits are lost rapidly upon drug discontinuation - a critical limitation in a chronic disease requiring lifelong management. (3) Fibrosis improvement in NASH: The inability of semaglutide to improve hepatic fibrosis despite achieving NASH resolution - contrasted with BMS producing fibrosis improvement in 46-47% of patients - represents a mechanistically important differentiator with direct implications for liver-related mortality. Fibrosis stage, not inflammation alone, determines NASH prognosis. (4) Cardiovascular mortality (absolute risk reduction): The 52% relative MACE risk reduction for BMS versus GLP-1 RAs in direct head-to-head analysis represents an incremental benefit over and above the intrinsic cardioprotection of GLP-1 RAs themselves. The mechanism likely involves weight-independent improvements in endothelial function, neurohormonal signalling, and visceral adiposity reduction beyond that achievable pharmacologically. (5) Metabolic reprogramming depth: BMS induces a constellation of weight-dependent and weight-independent metabolic changes - altered gut peptide secretion, bile acid composition, gut microbiota, and adipokine profiles - that collectively produce a degree of metabolic normalisation not achievable through receptor agonism at a single endocrine target.

Towards a Complementary and Sequential Paradigm

The false dichotomy of "surgery versus drugs" should be replaced with a more nuanced framework of complementary and sequential use. GLP-1 RAs and dual agonists can serve as: (1) pre-operative agents to reduce perioperative risk in patients with very high BMI; (2) post-operative adjuncts to prevent weight regain or optimise residual metabolic abnormalities; (3) primary therapy in patients who decline surgery, have contraindications, or present with early T2DM or obesity without surgical indications; and (4) bridging therapy while awaiting surgical access in healthcare systems with limited capacity. The emerging literature on GLP-1 RA use following BMS warrants prospective evaluation in dedicated trials.

Clinical and Guideline Implications

The evidence synthesised in this review is sufficient to support three specific clinical guidance updates: (1) BMS should be positioned in national and international guidelines as a first-line therapeutic option - not a last resort - for patients with obesity (BMI ≥30 kg/m²) and established T2DM, particularly those with diabetes duration <8 years, high cardiovascular risk, or biopsy-confirmed NASH with F2-F3 fibrosis; (2) Incretin therapies (particularly tirzepatide) should be recognised as effective alternative first-line therapy for patients with obesity and early T2DM or prediabetes who decline or are deferred from surgery; (3) Combined/sequential surgical and pharmacological strategies warrant prospective RCT evaluation as a formally recognised management paradigm.

Limitations

Several limitations merit acknowledgement. The primary pharmacological trials (STEP-1, SURMOUNT-1) enrolled predominantly non-diabetic participants and are not directly comparable with surgical T2DM RCTs. The absence of a head-to-head RCT comparing tirzepatide with BMS in matched T2DM populations represents the most important current evidence gap. Statistical heterogeneity across observational cohort studies is substantial (I²=78% in the Chadwick meta-analysis [13]), reflecting differences in populations, surgical procedures, and GLP-1 RA agents. All pharmacological comparator data for MACE and mortality are based on observational designs after direct RCT comparison was unavailable. Long-term incretin pharmacotherapy data beyond five years are not available, precluding comparison with 10-12-year surgical outcomes data. These conclusions are consistent with an independent decade-long systematic review of RCT evidence comparing bariatric surgery with medical therapy, which similarly reported consistent surgical superiority in glycaemic control, weight reduction, quality of life, and renal protection, albeit without a GLP-1 RA-specific or MACE-focused comparison [20].

## Conclusions

This head-to-head systematic review of 14 studies encompassing 25,566 participants demonstrates that BMS maintains decisive superiority over current incretin-based pharmacotherapy across all primary outcome domains in adults with obesity and established T2DM. BMS achieves T2DM remission at rates pharmacological agents cannot approach, delivers more durable weight loss, reduces MACE by 52% over GLP-1 RAs in direct comparison, produces NASH resolution with concurrent fibrosis improvement that semaglutide cannot replicate, and yields greater long-term quality of life improvements. Incretin therapies - particularly the dual GIP/GLP-1 agonist tirzepatide - represent genuinely transformative advances in obesity pharmacotherapy that approach surgical weight outcomes at 72 weeks and demonstrate meaningful cardiovascular benefit. These agents expand therapeutic options for patients who decline, defer, or are contraindicated for surgery, and may serve important roles as bridge or adjunct therapies in a combined treatment paradigm.

The clinical community and guideline bodies should move beyond the binary of "surgery versus drugs" towards evidence-based stratification: surgery as first-line for established T2DM with high cardiovascular or hepatic risk; incretins for early T2DM, prediabetes, or patients preferring non-surgical management; and combined sequential strategies as an emerging paradigm requiring prospective evaluation. The most urgent unmet research need is a head-to-head RCT comparing tirzepatide 15 mg with RYGB or SG in matched adults with T2DM and obesity, with hard cardiovascular, histological, and long-term metabolic outcomes as co-primary endpoints.
